# Gonorrhea, Chlamydia and HIV incidence among female sex workers in Cotonou, Benin: A longitudinal study

**DOI:** 10.1371/journal.pone.0197251

**Published:** 2018-05-10

**Authors:** Souleymane Diabaté, Annie Chamberland, Nassirou Geraldo, Cécile Tremblay, Michel Alary

**Affiliations:** 1 Axe Santé des populations et pratiques optimales en santé, Centre de recherche du CHU de Québec–Université Laval, Québec, Québec, Canada; 2 Département de médecine sociale et préventive, Université Laval, Québec, Québec, Canada; 3 Département d’infectiologie et santé publique, Université Alassane Ouattara, Bouaké, Côte d’Ivoire; 4 Centre de recherche du Centre hospitalier universitaire de Montréal, Montréal, Québec, Canada; 5 Dispensaire IST, Centre de santé de Cotonou I, Cotonou, Bénin; 6 Institut national de santé publique du Québec, Québec, Québec, Canada; Institute of Tropical Medicine, BELGIUM

## Abstract

Female sex workers (FSWs) continue to carry a heavy burden of sexually transmitted infections (STI). For prevention purposes, there is a need to identify most-at-risk subgroups among them. The objective of this longitudinal cohort study conducted at *Dispensaire IST*, Cotonou, Benin, was to assess *Neisseria gonorrhoeae* (NG) / *Chlamydia trachomatis* (CT) incidence and determinants; and HIV incidence among FSWs in presence of STI/HIV risk reduction activities. Overall, 319 adult FSWs were followed quarterly from September 2008 to March 2012. NG/CT were detected from endocervical swabs by Amplified DNA Assays employing Strand displacement amplification technology. HIV testing was done on capillary blood using two consecutive rapid diagnostic tests. Anderson-Gill proportional hazard models (HR) were used to determine factors independently associated with NG/CT incidence. The majority of FSWs were HIV-negative (188, 58.9%). There were 6 HIV seroconversions among these 188 HIV-negative women. HIV incidence (95% Confidence interval, CI) was 1.41 (0.28–2.54) seroconversions per 100 person-years at risk (PYAR): 6 events / 425.1 PYAR. Sixty-two out of 319 women experienced 83 new episodes of NG/CT for an overall incidence rate (95% CI) of 10.8 (8.17–13.88) events / 100 PYAR. From month-24 onwards, HIV-positive women (treated: HR (95%CI): 4.2 (1.60–10.77); untreated: HR (95%CI): 4.2 (1.59–11.49) were more likely to acquire NG/CT compared to HIV-negative FSWs. Longer duration in sex work (>2 years: HR; 95%CI: 0.4 (0.22–0.72)) was protective against NG/CT. Refusal by clients (55.8%) was the main reason for non-condom use. Enrolling women from one clinic (*Dispensaire IST*) may have impaired generalizability of the findings. New NG/CT/HIV infections were observed among FSWs notwithstanding ongoing prevention interventions. To eliminate HIV transmission among FSWs, STI/HIV control programs need to promote women’s empowerment and address vulnerability to infection of HIV-positive FSWs.

## Introduction

Female sex workers (FSWs) are generally undereducated, socially marginalized and exposed to violence [1, 2]. Their low socio-economic power hinders their authority to negotiate condom use with their clients and boyfriends [3] and exposes them to sexually transmitted infections (STI), including HIV [4]. FSWs are 13.5 times more likely to be HIV-positive (HIV+) compared to other women in low- and middle-income countries [5, 6]. In West Africa, up to 32% of new HIV infections are related to sex work [5]. In Benin, HIV prevalence is estimated at 1.1% in the general population while it reaches 20%-25% among FSWs [5, 7]. The disproportionate burden of HIV infection among FSWs is in accordance with a relatively high STI prevalence [4]. STI increase HIV infectiousness and susceptibility through different biological mechanisms [8]. Controlling STI transmission could contribute to the global fight against the HIV epidemic among FSWs and among the general population in contexts where HIV transmission is predominantly heterosexual [9, 10]. The identification of the characteristics of FSWs who have a high probability of acquiring STI repeatedly could contribute to improve strategies addressing STI/HIV acquisition in this population and limit onward transmission to their clients and the general population.

The aims of this cohort study conducted at *Dispensaire IST* in Cotonou (Benin) were to investigate the incidence and determinants of *Neisseria gonorrhoeae (*NG) and *Chlamydia trachomatis* (CT) taking into account multiple occurrences of these infections in some subjects, as well as HIV incidence in presence of STI/HIV risk reduction activities that are in place at Dispensaire IST: communication for behavioural change, condom distribution, promotion of correct and consistent condom use, and improvement of STI/HIV knowledge. *Dispensaire IST* is the main clinic dedicated to FSWs in Cotonou, the largest city of Benin. It offers monthly clinical check-ups with free STI treatment based on the syndromic approach to all FSWs as well as antiretroviral therapy (ART) to HIV+ ones.

## Methods

FSWs were consecutively recruited and followed between September 2008 and March 2012. All women (age≥18 years) who attended the clinic were invited to participate. Information on socio-demographic characteristics (education, marital status, dependent children), type of sex and sexual behaviour (vaginal, oral and anal sex, concurrent partnerships, consistent condom use (CCU)), type and number of sexual partners (regular, occasional, and commercial sex partners) was obtained through face-to-face interviews using standardized questionnaires administered by trained investigators. Clinical data including syndromic diagnosis of NG/CT were obtained at enrolment, at every scheduled three-monthly visit and at any inter-current visit (monthly clinical check-up) through physical, bimanual vaginal and speculum examinations by a physician. Syndromic diagnoses of NG/CT corresponded to clinical signs of pelvic inflammatory disease (cervical motion tenderness and/or lower abdominal pain during the bimanual palpation of the pelvis) or mucopus from the cervix or a yellow swab or blood on the endocervical swab that was collected for laboratory testing. At enrolment, women underwent laboratory screening for NG/CT/HIV. Thereafter; laboratory testing for NG/CT was done twice per year. Women who were HIV-negative (HIV-) at study entry were retested quarterly. NG/CT infections (whether based on syndromic diagnosis or laboratory testing) were treated orally with ciprofloxacin (500 mg in a single dose) and doxycycline (100 mg twice daily for seven days) as recommended in Benin during the study period. CD4 count was obtained quarterly for HIV+ women by means of flow cytometry. Those who met the 2006-World health organisation (WHO) criteria for ART initiation (CD4 count ≤200/mm^3^ or HIV clinical stage IV or stage III plus CD4 count <350/mm^3^) prevailing in Benin during the study period were treated [11]. The other HIV+ women remained untreated until they met the criteria. During the study period peer-educators and community workers regularly met FSWs for training/discussion sessions to demonstrate correct condom use, to promote its consistent use and to improve FSWs’ knowledge about the warning signs, consequences, and treatment options of HIV/STI. They distributed condoms for free at each session and promoted monthly clinical check-ups (free of charge) at the study centre. The training/discussion sessions were organized on a daily basis at brothels and other sex work venues in Cotonou, and at the *Dispensaire IST*. Brothels are private houses where FSWs live and work. All brothels and hot spots in Cotonou were identified using an update of a previous mapping realized by a non-governmental organization our study team has been working with for more than 15 years [10]. In addition to the training/discussion sessions, there was also routine (monthly) clinical check-ups/treatment of NG/CT and other STI at *Dispensaire IST*. FSWs were examined by a general practitioner and they were provided with free STI treatments when a syndromic STI diagnosis was established. Women who had a positive laboratory test also received NG/CT treatment for free. During routine visits for clinical check-ups at the clinic, women were generally grouped by brothel or hot spot. Primary care services for other common health problems were also available at *Dispensaire IST*.

At the time of physical examination, the physician collected an endocervical swab that was tested by the BD Probe TecTM ET *Chlamydia trachomatis* and *Neisseria gonorrhoeae* Amplified DNA Assays (Becton-Dickinson Inc., Sparks, MD, USA) employing Strand displacement amplification (SDA) technology. Two consecutive HIV rapid diagnostic tests, Determine® HIV 1/2 test (Abbott Diagnostic Division, Hoofddorp, The Netherlands) confirmed with Genie II® HIV1/2 (Bio-Rad, Marnes-la-Coquette, France) or SD Bioline HIV1/2 (Standard Diagnostics, Inc., Gyeonggi-do, South Korea) were used for HIV testing on capillary blood.

All new NG/CT infections diagnosed through laboratory testing after study entry were included in the analyses of incidence rates (numerator). At-risk period (denominator): a woman with any prior infection was considered at risk of a new episode from day-7 after the diagnosis to the next positive test or to the end of follow-up, whichever occurred first. The seven-day window corresponds to the treatment period during which women were assumed not to be at risk. We also subtracted seven days from the denominator for women who received treatment based on syndromic diagnosis. For HIV infection, it was postulated that seroconversion occurred at the mid-point between the follow-up visit during which the test was positive and the previous visit where it was negative.

NG/CT/HIV incidence rates were obtained by dividing the number of events by the person-years at risk (PYAR). A recurrent event and proportional means model was used to take into account the within subject correlation due to multiple measurements and the fact that some subjects have experienced more than one event. Bootstrapping with 1000 repeated re-sampling of the data was used to calculate the 95% CI. Univariate and multivariate Anderson-Gill proportional hazard models (HR) were used to determine factors independently associated with NG/CT occurrences (p-value < .05). Robust sandwich variances estimates of the standard errors taking into account dependency due to repeated measurements of the issue of interest were used to obtain p-values [12]. We tested the proportionality assumption of the hazard ratios. Baseline variables that were assessed for their independent association with NG/CT incidence included HIV serostatus, duration in sex work, place of work, CCU, country of origin, marital status, number of clients, and age. They were considered for inclusion in these analyses based on our research experience in the sex work milieu in Benin and on literature review [10, 13–18]. Finally, we performed a post hoc analysis of the performance of the syndromic diagnosis. All statistical analyses were carried out using SAS 9.3 (SAS Institute, Cary, North Carolina, USA).

Women who agreed to participate in the study provided informed written consent. The study was approved by the *Comité National Provisoire d’Éthique de la Recherche en Santé* (037/CNPERS/SA) and the Ministry of Health of Benin (3721/MS/DC/SGM/DRS/SCI/SA), and by the research ethics committees of the Centre hospitalier universitaire de Montréal (08–116) and the CHU de Québec–Université Laval (2008HSS-263-04) in Canada.

## Results

### Baseline characteristics

Overall, 396 FSWs were enrolled but 3/52 HIV+/treated, 13/95 HIV+/untreated and 61/249 HIV- women did not return for follow-up visits after being enrolled. Those women did not contribute to PYAR. In addition, 16 women were considered as lost to follow-up at the end of the study. All these women were all quite similar to their counterparts who remained in the study according to baseline characteristics and NG/CT prevalence. As presented in Table 1, the sample analyzed was composed of 319 women with at least one follow-up visit (49 HIV+/treated, 82 HIV+/untreated and 188 HIV-).

**Table 1:** Baseline characteristics of 319 female sex workers, Cotonou, Benin, 2008–2012.

Characteristics	Number (%)
Nationality	
*Benin*	123 (38.5)
*Togo*	74 (23.2)
*Nigeria*	66 (20.7)
*Ghana*	49 (15.4)
*Others*	7 (2.2)
Age in years	
*18–24*	48 (15.1)
*25–34*	112 (35.1)
*≥ 35*	159 (49.8)
Education	
*Not educated*	107 (33.5)
*Primary*	126 (39.5)
*Secondary*	79 (24.8)
*Postsecondary*, *university*	7 (2.2)
Marital status	
*Married*	21 (6.6)
*Single*	102 (32.0)
*Divorced*	149 (46.7)
*Widowed*	47 (14.7)
Place of work ^a^	
*Brothel*	126 (39.6)
*Street*	76 (23.9)
*Others (bars*, *hotels*, *etc*.*)*	116 (36.5)
Duration in sex work, months; median (IQR)	36 (16–60)
Number of clients, last 7 days of work; median (IQR)	13 (5–26)
Number of sexual intercourses, last 3 days; median (IQR)	5 (1–11)
Consistent condom use with clients (last 7 days of work)	246 (77.1)
Condom use with last client	279 (87.5)
HIV/treatment status	
*HIV positive*, *treated*	49 (15.4)
*HIV positive*, *not treated*	82 (25.7)
*HIV negative*	188 (58.9)
Ever had anal sex (any partner)	16 (5.0)
Ever had oral sex (any partner)	62 (19.4)
NG prevalence	13 (4.1)
CT prevalence	9 (2.8)
NG/CT ^b^ prevalence	21 (6.6)
CD4 cell count/mm^3^; median (IQR) ^c^	389 (215–583)
Viral load; median (IQR) ^c^	8153 (154–31321)

Abbreviations: CD4, CD4 T lymphocytes; IQR, Interquartile range; CT, Chlamydia trachomatis; FSWs, female sex workers; IQR, interquartile range; NG, Neisseria gonorrheae

a The differences in total number is due to non-response (n = 1)

b Infection by either NG and/or CT

c HIV-Positive female sex workers.

The majority of these 319 women were HIV- (58.9%). The most frequent country of origin was Benin (38.6%) followed by Togo (23.2%). More than half of the women were divorced or widowed. About one third never attended school and about 40% were working in brothels. During the last seven days of work, -CCU- with clients was reported by around three quarters of the women: 71.4% for HIV+/treated, 74.4% for HIV+/untreated and 79.8% for HIV- women. Refusal of condoms by the partner was the major reason reported for the last unprotected sex with a client (55.8%). Except for non-negotiation for condom use by the woman herself (11.6%), other reasons were diverse and were accounting for <4% each: conviction that the client was HIV-, decision to marry the client, condoms use reduces sexual pleasure, and condom stock-outs. At baseline, 4.1% and 2.8% of the women were diagnosed with NG and CT, respectively. Two women were concomitantly infected with both pathogens.

Median (interquartile range (IQR)) duration in the study was 1.95 (0.43–3.49) years. There was a weak correlation between age and duration in sex work (Spearman’s rank correlation coefficient = 0.43).

### HIV incidence

Of the 188 HIV- women who completed at least one follow-up visit, HIV incidence (95% CI) was 1.41 (0.28–2.54) seroconversions /100 PYAR (Table 2).

**Table 2:** Incidence of *Neisseria gonorrhoeae*, *Chlamydia trachomatis* and HIV infection in a cohort of 319 female sex workers in Cotonou, Benin, 2008–2012.

	All women (N = 319)		HIV-positive, treated (N = 49)		HIV-positive, untreated (N = 82)		HIV-negative (N = 188)	
Incident ^a^	Rate (n/PYAR)	95% CI	Rate (n/PYAR)	95% CI	Rate (n/PYAR)	95% CI	Rate (n/PYAR)	95% CI
NG	8.9 (68/765.73)	6.64–11.20	11.2 (19/169.96)	5.96–16.98	10.7 (19/177.49)	5.71–16.52	7.2 (30/418.28)	4.97–9.78
CT	2.1 (16/766.32)	0.91–3.43	0.6 (1/170.23)	0.00–1.86	3.9 (7/177.63)	0.60–8.21	1.9 (8/418.47)	0.48–3.60
NG/CT	10.8 (83/765.56)	8.17–13.88	11.8 (20/169.94)	6.19–17.85	14.1 (25/177.47)	7.34–22.16	9.1 (38/418.14)	6.08–12.23
**HIV incidence rate** ^**b**^							1.41 (6/425.102)	0.28–2.54

Abbreviations: CI, Confidence interval; PYAR, Person-years at risk; NG, *Neisseria gonorrhoeae*; CT, *Chlamydia trachomatis*; NG/CT, infection by either NG and/or CT

^a^ Occurrences of NG/CT (new events / 100 PYAR), some women had more than one new episode

^b^ Number of sero-conversions / 100 PYAR

### NG/CT incidence

Sixty-two (62) women experienced 83 new episodes of NG/CT according to laboratory diagnosis (48 women had 1 episode, 11 had 2, 2 had 4 and 1 had 5). Median duration between NG/CT episodes was 363 (IQR: 179–593) days. The total number of PYAR accumulated was 765.56 and the overall incidence rate (95% CI) for infection by either *N*. *gonorrhoeae* or *C*. *trachomatis* (NG/CT) was 10.8 (8.17–13.88) events /100 PYAR (Table 2). During follow-up, a syndromic diagnosis was made by the clinician 579 times in a total of 2409 visits. When considering the 1253 visits where both syndromic assessment (344 syndromic diagnoses of NG/CT) and laboratory testing (83 positive tests for either NG or CT) were done, the intrinsic and predictive values of the syndromic approach compared to laboratory testing were: sensitivity (31/83; 37.4%), specificity (857/1170; 73.3%), positive predictive value (31/344; 9.0%), and negative predictive value (857/909; 94.3%).

When determining the predictors of NG/CT new infections based on laboratory results, there was some evidence of non-proportional hazards for HIV serologic and treatment status (p for proportionality = 0.05 (univariate model) and 0.08 (multivariate model; Table 3)). Model-2 was performed to account for that non-proportionality at month-24 of follow-up (Table 3).

**Table 3:** Univariate and multivariate Anderson-Gill proportional hazard models for factors associated with new NG/CT infections in a cohort of 319 female sex workers in Cotonou, Benin, 2008–2012.

Variables ^a^ | Model	NG/CT incidence rate per 100 PYAR (number/ PYAR)	Univariate			Multivariate		
		Model 1: No time strata	Model 2: time strata^b^		Model 1: No time strata	Model 2: time strata^b^	
			≤24 months	>24 months		≤24 months	>24 months
		HR (95%CI)	HR (95%CI)	HR (95%CI)	HR (95%CI)	HR (95%CI)	HR (95%CI)
HIV/treatment status ^c^							
HIV-	9.1 (38/418.14)	1	1	1	1	1	1
HIV+/Treated ^d^	11.8 (20/169.94)	1.3 (0.71–2.31)	0.8 (0.35–1.78)	3.0 (1.11–7.99)	1.9 (1.04–3.39)	1.2 (0.51–2.67)	4.2 (1.60–10.77)
HIV+/Untreated ^e^	14.1 (25/177.47)	1.5 (0.84–2.86)	1.0 (0.54–1.90)	4.1 (1.33–12.37)	1.9 (1.10–3.26)	1.3 (0.73–2.45)	4.2 (1.53–11.49)
Duration in sex work in months							
25+	8.0 (35/440.40)	1			1	1	
0–12	19.3 (29/150.39)	2.5 (1.30–4.64)			2.5 (1.42–4.56)	2.5 (1.39–4.43)	
13–24	11.0 (17/154.96)	1.4 (0.75–2.57)			1.4 (0.76–2.45)	1.4 (0.77–2.54)	
Place of work							
Brothels	5.5 (16/290.89)	1			1	1	
Street	13.5 (27/200.40)	2.5 (1.24–4.97)			1.9 (0.92–3.78)	1.8 (0.89–3.59)	
Bars, hotels, and other	14.7 (40/271.82)	2.7 (1.37–5.32)			2.4 (1.23–4.80)	2.4 (1.19–4.68)	
Consistent condom use							
Yes	9.7 (67/691.76)	1			1	1	
No	22.9 (16/69.94)	2.4 (1.24–4.59)			2.0 (1.11–3.72)	2.0 (1.11–3.72)	
Country of origin							
Other	7.3 (33/451.92)	1					
Benin	15.9 (50/313.64)	2.2 (1.32–3.67)					
Marital status							
Married or single	8.0 (22/276.80)	1					
Widowed or divorced	12.5 (61/488.75)	1.6 (0.93–2.64)					
Age at inclusion (years)							
≥35	10.8 (46/427.52)	1					
25–34	10.4 (26/251.29)	1.0 (0.54–1.74)					
18–24	12.7 (11/86.75)	1.2 (0.63–2.28)					
Number of clients							
No client	6.7 (5/75.11)	1					
1–12	12.1 (48/395.81)	1.8 (0.72–4.56)					
13+	10.3 (30/290.79)	1.6 (0.61–4.06)					

Abbreviations: CI, Confidence interval; CT, *Chlamydia trachomatis*; HR, Hazard ratio; NG, *Neisseria gonorrhoeae*; NG/CT, infection by either NG and/or CT PYAR, Person-year at risk

^a^ Differences in numbers due to missing values

^b^ From enrolment to month-24 versus >24 months of follow-up

^c^*P*, for proportionality = 0.05 (univariate) and 0.08 (multivariate)

^d^*P* for differences between periods in model 2 = 0.03 (univariate) and 0.03 (multivariate)

^*e*^ *P* for differences between periods in model 2 = 0.02 (univariate) and 0.03 (multivariate)

In multivariate models, NG/CT infections were more likely to occur in HIV+ women after two years of follow-up compared to their HIV negative counterparts. Longer duration in sex work (>2 years) and CCU were protective against NG/CT while working on streets, in bars and hotels (compared to brothels) was a risk factor. Marital status, country of origin, number of clients and age were not independently associated with the outcome of interest and they did not change significantly other associations (variation between crude and adjusted HR <10%).

Crude incidence rates of NG/CT were computed to better understand departure from proportionality from enrolment to month-24 versus >month-24 of follow-up, with regard to HIV serologic and treatment status (Table 4). In HIV- women, we observe a sharper decline in NG/CT incidence when women who acquired HIV during follow-up were excluded from the baseline HIV- group (as treated pattern) compared to when they were maintained in the baseline HIV- group (intention to treat pattern): ~2-fold versus ~1.5-fold decrease in NG/CT incidence, respectively. At the same time, there was an increase in crude incidence rates of NG/CT in HIV+/untreated women: ~1.5-fold increase for the «intention to treat» pattern and ~2-fold increase for the «as treated» pattern that accounted for switches from HIV- to HIV+/untreated group and from HIV+/untreated to HIV+/treated group.

**Table 4:** Variation in crude NG/CT incidence rates in a cohort of 319 female sex workers, in Cotonou, Benin, 2008–2012.

Type of analyses	Study groups	Incidence rate (95% CI)^a^		Variation (before and after month-24)
		≤ 24 months	> 24 months	
Initial group^b^	HIV-	11.5 (11.11–11.89)	7.9 (7.43–8.37)	~ 1.5-fold ↓
	HIV+/Treated	8.3 (7.69–8.91)	16.6 (15.57–17.63)	~ 2-fold ↑
	HIV+/Untreated	9.8 (9.27–10.33)	14.4 (13.46–15.34)	~ 1.4-fold ↑
Switches between groups^c^	HIV-	10.9 (10.52–11.28)	5.3 (4.91–5.69)	~ 2-fold ↓
	HIV+/Treated	8.6 (8.00–9.20)	15.6 (14.72–16.48)	~ 1.8-fold ↑
	HIV+/Untreated	11.1 (10.92–11.28)	21.4 (20.13–22.67)	~ 2-fold ↑

↓, decrease

↑, increase

^a^ per 100 person-years at risk

^b^ Groups as constituted at enrolment with analyses not taking into account switches between groups during follow-up

^c^ Analyses taking into account switches between groups. Switches occurred from HIV- to HIV+/untreated (mainly) and to HIV+/ treated (secondarily), and from HIV+/untreated to HIV+/treated; CI, Confidence interval; CT, *Chlamydia trachomatis*; NG, *Neisseria gonorrhoeae*; NG/CT, infection by either NG and/or CT

## Discussion

New NG/CT/HIV infections were observed among FSWs notwithstanding ongoing promotion of correct and CCU, free condom donation, quarterly lab screenings, and free STI treatment. NG/CT incidence rate was 10.8 (8.17–13.88) events /100 PYAR. From month-24 onwards, HIV-positive women (treated: HR (95%CI): 4.2 (1.60–10.77); untreated: HR (95%CI): 4.2 (1.59–11.49)) were more likely to acquire NG/CT compared to HIV-negative FSWs. Shorter duration in sex work, inconsistent condom use and not working in brothels were risk factors for new NG/CT infections. HIV incidence was 1.41 (0.28–2.54) seroconversions /100 PYAR.

Even though NG/CT prevalence among FSWs has declined steadily over the past two decades in Benin due to a mix of structural, clinical and behavioural change interventions [10], FSWs continue to experience re-infections. In line with previous reports, non-condom use was a stronger predictor of new NG/CT infections and was mainly due to refusal by clients [15]. Unfortunately, the need for income and fear of losing clients weakens the capacity of FSWs to impose condoms even though they are aware of the potential consequences of unprotected sex [19]. Some clients pay high amounts of money for unsafe sex. A 43% increase in mean payment for unsafe sex was reported in Zimbabwe [19]. Violence perpetrated by clients is also associated with inconsistent condom use among FSWs [20]. According to these observations highlighting clients’ responsibility in unsafe sex, it will be toilsome for STI/HIV control programs to achieve CCU in the sex work milieu by targeting only FSWs while leaving aside their clients: In spite of routine STI services and care, and awareness-raising discussions implemented sometimes on a daily basis as done in this study, FSWs fail to achieve CCU with their clients and they continue to pay a high toll to STI/HIV [21]. More than half of the unprotected sexual intercourses were explained by clients’ behaviour i.e. refusal of condoms, although the reasons given by FSWs to explain the lack of condom us may be prone to social desirability bias. So, for condom use in the sex work milieu to become universal, programmes need to include clients in addition to the FSWs themselves [22], and to promote methods under women’s control like female condoms. Differences between outcomes’ definition, first versus multiple occurrences of NG/CT, make it difficult to compare incident rates of these STI across studies [13, 14].

A novel finding in this study is the fact that after 24 months of follow-up, HIV+ women were more likely to be re-infected with NG/CT compared to their HIV- fellows. Since condom use was not consistent, this difference could have partly been explained by less motivation to use condoms for their own protection and/or by an increase in sexual activity due to enhancement in HIV+ women’s health status over time [23, 24]. ART among HIV+/treated FSWs, and chemoprophylaxis or management of co-morbidities among HIV+/untreated ones may have contributed to an increase in sexual activity among some women who were not fully active before care due to more precarious health. In Cameroon, time since ART initiation was found to be significantly associated with sexual activity [24]. However, in this study, resumption or increase in sexual activity among HIV+ women can hardly explain higher infection rates from month-24 onwards. Departure from proportionality in NG/CT risk is probably related to a decreased risk in the reference group (HIV-): during follow-up, most-at-risk HIV- women have seroconverted and have moved to HIV+ groups where they have maintained their risky behaviour. This hypothesis is supported by the results depicted in Table 4: i) the protective effect observed over time among HIV- women is much stronger when those who seroconvert are excluded from the analyses (2-fold decrease from 10.9 (10.52–11.28) to 5.3 (4.91–5.69)) /100 PYAR compared to when analyses include all women who were HIV- at enrolment (1.5-fold decrease from 11.5 (11.11–11.89) to 7.9 (7.43–8.37)) /100 PYAR; ii) conversely, the risk of acquiring NG/CT among HIV+/untreated women is much stronger when switches between groups are considered. These observations highlight the importance of sustaining behaviour change communication for FSWs newly diagnosed with HIV infection in particular and for all HIV+ FSWs in general. These observations also support the necessity of new strategies for HIV and STI control such as early universal access to antiretroviral therapy, irrespective of CD4 count, and Pre-exposure prophylaxis (PrEP) for key populations [25]. Indeed, PrEP could be implemented among FSWs who are particularly engaged in risky behaviours like inconsistent condom use and who are consequently at higher risk of acquiring STI and HIV infections.

Sex work not related to brothels was strongly associated with NG/CT infections during follow-up. This result was expected as women working outside brothels are more mobile, moving from one place to another looking for clients and to avoid police harassment [15, 16]. This makes it relatively hard to reach these women and to regularly include them in interventions. In contrast, brothels are well-identified houses that are routinely visited by peer-educators and community workers. In accordance with less exposure to prevention and care activities, shorter duration in sex work was independently associated with NG/CT infections. Shorter duration in sex work implies reduced exposure to outreach activities and limited experience in negotiating condom [16]. Shorter duration in sex work results in a higher turnover in the sex work milieu [15, 16]. In this study, one-fourth of the women had been involved in sex work for at most 12 months.

HIV incidence rate in this study was two to three times lower compared to incidence rates (95% CI) reported in Rwanda (3.5 (1.60–5.40) /100 PYAR) and Uganda (3.66 (2.71–4.96) /100 PYAR) [26, 27], and six times lower than in Zimbabwe (9.8 (7.1–15.9) /100 PYAR) [28]. Numerous and complex reasons including the fact that HIV epidemic is more generalized in eastern Africa as compared to western Africa and the STI/HIV prevention and care package provided in the context of this study may explain these differences. Recently, Traoré IT et al. found no seroconversion in presence of prevention activities among 321 HIV-uninfected FSWs who completed 409 person-years of follow-up in Burkina Faso [29]. In 2015, HIV prevalence in the general adult population was slightly higher in Benin (1.1%) than in Burkina Faso (0.8%), two neighbouring countries [5, 7]. In Benin, FSWs are much more affected. In 2012 during the study period, HIV prevalence was estimated at 21% among FSWs in Benin compared to 16.4% in Burkina Faso [5]. Hence, differences in HIV incidence rates in this study and that of Traoré IT et al. [29] could result from differences in HIV dynamic among FSWs. In a randomized control trial on the microbicide cellulose sulphate conducted between 2005 and 2007 and that showed negative results, HIV incidence was 5.9/100 woman-years (unpublished local data from the study) [30]. Compared to the incidence found in the present study (1.4/100 person-years) conducted in the same settings, there is an encouraging decrease of more than 80%. In spite of this decline, HIV incidence remains 15.7 to 20.1 times higher in FSWs than in the general population: according to UNAIDS annual estimates, the HIV incidence rate (uncertainty range) among the adult general population was 0.08 (0.05–0.11) /100 PYAR in 2008 and 0.06 (0.04–0.09) /100 PYAR in 2016 in Benin [7]. Continuing transmission at higher rates among FSWs indicates once again that behaviour change counselling, condom promotion, and STI diagnosis/treatment targeting only FSWs (as done in the context of this study) are not sufficient to interrupt HIV transmission.

Strengths of this prospective cohort study included: i) the ability to determine the limited scope of non-inclusive preventive interventions that target FSWs while leaving aside their clients who contribute significantly to unsafe sex; and ii) being able to identify continuing risk-taking among HIV- FSWs who seroconverted by testing the proportionality assumption of the hazard ratios.

Limitations of this study encompass the fact that *Dispensaire IST* is the main public clinic that has been offering routine STI services adapted to FSWs needs for more than two decades in Cotonou and its suburbs. As such, our convenience sample is likely to be quite representative of all FSWs of Cotonou and its suburbs who seek STI care. Underestimation of HIV incidence is expected if most-at-risk women tended to fall out of the study. However, assessment of baseline characteristics revealed that women who did not complete at least one follow-up visit and those lost to follow-up were generally similar to their counterparts. Some women have received NG/CT treatment based only on syndromic diagnoses. Since syndromic diagnoses were done at least monthly (monthly clinical check-ups and quarterly scheduled visits) and NG/CT laboratory testing every six months, this could have underestimated the number of incident cases detected through laboratory testing if those treated women had recovered before undergoing laboratory testing. In spite of that, only a weak effect on the results is expected due to the low positive predictive value (9.0%) of the syndromic approach. Indeed, most women treated through the syndromic approach were in fact not NG/CT infected. On the other hand, the syndromic approach has captured only 37.4% of NG/CT infected women. It should be noted that comparable values of sensitivity and specificity of this same syndromic approach were reported in our study population in 2002 [31]. However, the positive predictive value has declined from 38.3% in 2002 to 9.0% in the current study due to a decline in NG/CT prevalence from 24.5% in 2002 to 6.6% (83 NG/CT cases / 1253 visits) in the current study [31]. The limited capacity of our syndromic approach to accurately identify NG/CT infected women (sensitivity of 37.4%) is similar to the situation reported by Francis et al. [32] who showed that most curable STI in vulnerable Tanzanian women were missed by syndromic management. They recommended the use of accurate and affordable rapid-point-of-care tests for STI management [32]. Women experiencing NG/CT infections were assumed to be cured after seven days of treatment. This could have overestimated the episodes of NG/CT in case some women were not actually cured from previous events. However, this must be the exception since women were tested every six months (180 days) and the median duration between NG/CT episodes among women with recurrent infection was 363 (IQR: 179–593) days. The relatively low HIV incidence rate prevents us from appraising the determinants of HIV acquisition and comparing them with those of NG/CT infections. Finally, the data were collected more than five years ago and the dynamic of STI/HIV could have evolved since then.

In light of the results of this study, one may suggest in depth research on: i) the capacity of current prevention methods to control STI transmission and to end the HIV epidemic in the coming decades as intended by the UNAIDS [33]; ii) the identification of combination STI and HIV prevention packages for fast and cost-effective control of these infections in vulnerable populations; iii) and the feasibility in different contexts of PrEP for most-at-risk sub-populations like FSWs. On their side, policy-makers and national programs in charge of STI an HIV control should promote free access to female condoms, rapid-point-of-care tests for STI management, and universal access to antiretroviral therapy. In the meantime, public health specialists should set up multidimensional behavioural interventions based on theoretical models that explain behaviour in order to achieve CCU among all FSWs including those working outside brothels and who are, thus, hard to reach. All these interventions should be implemented along with community empowerment.

## Conclusion

To eliminate transmission among FSWs in particular and in the sex work milieu in general, STI/HIV control programs should not target FSWs only. They should rather comprehensively encompass the whole sex work milieu to make it a safer environment with better respects of FSW’s human rights. In addition, it is necessary to consider the scale-up of early treatment for HIV-positive FSWs, PrEP for the most at-risk FSWs and methods under women’s exclusive control such as female condoms.

## Supporting information

S1 Data This file contains the minimal anonymized data set necessary to replicate the study findings. (XLSX)

S1 Codebook This file contains all codes related to the anonymized data set file. (DOCX)
