# Supplementary material for: Gonorrhea, Chlamydia and HIV incidence among female sex workers in Cotonou, Benin: A longitudinal study
Source: PLoS One. 2018 May 10;13(5):e0197251. doi: 10.1371/journal.pone.0197251 (PMC5945000; doi:10.1371/journal.pone.0197251)
Supplement: S1 Codebook — (DOCX) [file pone.0197251.s002.docx]

Codebook for the data file « data_file_Table_1.xlsx » (Table 1)

| Variable names | Variable descriptions | Codes |
| --- | --- | --- |
|  |  |  |
| unique | Subject identification number | Numeric |
| q103 | Nationality | 1 = Benin  2 = Ghana  3 = Nigeria  4 = Togo  5 = Others |
| age | Age in years | Numeric |
| age_3cat | Age in years (categories) | 1 = 18 - 24  2 = 25 - 34  3 = ≥ 35 |
| q105 | Education | 0 = Not educated  1 = Primary  2 = Secondary  3 = Postsecondary, university |
| q114 | Marital status | 1 = Married  2 = Single  3 = Divorced  4 = Widowed |
| q107 | Place of work | 1 = Brothel  2 = Home  3 = Bars  4 = Hotels  5 = Street  6 = Nightclub  7 = Others |
| duree | Duration in sex work, months | Numeric |
| q205 | Number of clients, last 7 days of work | Numeric |
| q219 | Number of sexual intercourses, last 3 days | Numeric |
| constante | Consistent condom use with clients (last 7 days of work) | 1 = Yes  2 = No |
| q211 | Condom use with last client | 1 = Yes  2 = No |
| groupe | HIV/treatment status | 1 = HIV positive, treated  2 = HIV positive, not treated  3 = HIV negative |
| anal | Ever had anal sex (any partner) | 1 = No  2 = Yes |
| hla9 | Ever had oral sex (any partner) | 1 = Yes  2 = No |
| gono | NG prevalence | 1 = Positive  2 = Negative  9 = No answer |
| chlam | CT prevalence | 1 = Positive  2 = Negative  9 = No answer |
| gonochlam | NG/CT prevalence | 1 = Positive  2 = Negative  9 = No answer |
| cd4_nb | CD4 cell count/mm³ | Numeric |
| charge_virale_copies | Viral load | Numeric |

Codebook for the data file « data_file_Table_2_gono.xlsx » (Table 2 for NG), « data_file_Table_2_chlam.xlsx » (Table 2 for CT),

« data_file_Table_2_vih_incident.xlsx » (Table 2 for HIV),

« data_file_Tables_2_3_and_4_gonochlam.xlsx » (Tables 2, 3 and 4 for NG/CT)

| Variable names | Variable descriptions | Codes |
| --- | --- | --- |
|  |  |  |
| unique | Subject identification number | Numeric |
| start0 | Start time in days for counting process style of input | Numeric |
| stop0 | Stop time in days for counting process style of input | Numeric |
| groupe | HIV/treatment status | 1 = HIV positive, treated  2 = HIV positive, not treated  3 = HIV negative |
| groupeinit | Initial group (HIV/treatment status, only for the table 4) | 1 = HIV positive, treated  2 = HIV positive, not treated  3 = HIV negative |
| duree_3cat | Duration in sex work, months (categories) | 1 = 0-12  2 = 13-24  3 = ≥ 25  9 = No answer |
| lieu | Place of work | 1 = Brothels  2 = Street  3 = Bars, hotels, and other  9 = No answer |
| constante | Consistent condom use with clients (last 7 days of work) | 1 = Yes  2 = No  9 = No answer |
| benin | Country of origin | 1 = Benin  2 = Other |
| statmat | Marital status | 1 = Married or single  2 = Widowed or divorced |
| age_3cat | Age in years (categories) | 1 = 18 - 24  2 = 25 - 34  3 = ≥ 35 |
| nbclidern2 | Number of clients, last 7 days of work | 1 = 1-12  2 = ≥ 13  8 = No client  9 = No answer |
| gono | NG | 0 = Negative  1 = Positive |
| chlam | CT | 0 = Negative  1 = Positive |
| gonochlam | NG/CT | 0 = Negative  1 = Positive |
| vih_incident | HIV | 0 = Negative  1 = Positive |
